# A G‐protein Subunit‐α11 Loss‐of‐Function Mutation, Thr54Met, Causes Familial Hypocalciuric Hypercalcemia Type 2 (FHH2)

**DOI:** 10.1002/jbmr.2778

**Published:** 2016-02-06

**Authors:** Caroline M Gorvin, Treena Cranston, Fadil M Hannan, Nigel Rust, Asjid Qureshi, M Andrew Nesbit, Rajesh V Thakker

**Affiliations:** ^1^ Academic Endocrine Unit, Radcliffe Department of MedicineUniversity of OxfordOxfordUK; ^2^ Oxford Molecular Genetics Laboratory, Churchill HospitalOxfordUK; ^3^ Department of Musculoskeletal Biology, Institute of Ageing and Chronic DiseaseUniversity of LiverpoolLiverpoolUK; ^4^ Sir William Dunn School of PathologyUniversity of OxfordOxfordUK; ^5^ Department of Diabetes and EndocrinologyNorthwest London NHS TrustLondonUK; ^6^ School of Biomedical SciencesUniversity of UlsterColeraineLondonderryUK

**Keywords:** DISORDERS OF CALCIUM/PHOSPHATE METABOLISM, PTH/VIT D/FGF23, PARATHYROID‐RELATED DISORDERS, CELL/TISSUE SIGNALING – ENDOCRINE PATHWAYS

## Abstract

Familial hypocalciuric hypercalcemia (FHH) is a genetically heterogeneous disorder with three variants, FHH1 to FHH3. FHH1 is caused by loss‐of‐function mutations of the calcium‐sensing receptor (CaSR), a G‐protein coupled receptor that predominantly signals via G‐protein subunit alpha‐11 (Gα_11_) to regulate calcium homeostasis. FHH2 is the result of loss‐of‐function mutations in Gα_11_, encoded by *GNA11*, and to date only two FHH2‐associated Gα_11_ missense mutations (Leu135Gln and Ile200del) have been reported. FHH3 is the result of loss‐of‐function mutations of the adaptor protein‐2 σ‐subunit (AP2σ), which plays a pivotal role in clathrin‐mediated endocytosis. We describe a 65‐year‐old woman who had hypercalcemia with normal circulating parathyroid hormone concentrations and hypocalciuria, features consistent with FHH, but she did not have CaSR and AP2σ mutations. Mutational analysis of the *GNA11* gene was therefore undertaken, using leucocyte DNA, and this identified a novel heterozygous *GNA11* mutation (c.161C>T; p.Thr54Met). The effect of the Gα_11_ variant was assessed by homology modeling of the related Gα_q_ protein and by measuring the CaSR‐mediated intracellular calcium (Ca^2+^_i_) responses of HEK293 cells, stably expressing CaSR, to alterations in extracellular calcium (Ca^2+^_o_) using flow cytometry. Three‐dimensional modeling revealed the Thr54Met mutation to be located at the interface between the Gα_11_ helical and GTPase domains, and to likely impair GDP binding and interdomain interactions. Expression of wild‐type and the mutant Gα_11_ in HEK293 cells stably expressing CaSR demonstrate that the Ca^2+^_i_ responses after stimulation with Ca^2+^_o_ of the mutant Met54 Gα_11_ led to a rightward shift of the concentration‐response curve with a significantly (*p* < 0.01) increased mean half‐maximal concentration (EC_50_) value of 3.88 mM (95% confidence interval [CI] 3.76–4.01 mM), when compared with the wild‐type EC_50_ of 2.94 mM (95% CI 2.81–3.07 mM) consistent with a loss‐of‐function. Thus, our studies have identified a third Gα_11_ mutation (Thr54Met) causing FHH2 and reveal a critical role for the Gα_11_ interdomain interface in CaSR signaling and Ca^2+^_o_ homeostasis. © 2016 The Authors. *Journal of Bone and Mineral Research* published by Wiley Periodicals, Inc. on behalf of American Society for Bone and Mineral Research (ASBMR).

## Introduction

**Figure 1:**
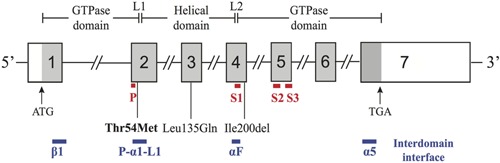
Schematic representation of the genomic organization of the human *GNA11* gene showing the locations of FHH2‐causing mutations. The *GNA11* gene consists of 7 exons with the start (ATG) and stop (TGA) codons located in exons 1 and 7, respectively. The GTPase domain (encoded by exon 1, 5' portion of exon 2, 3' portion of exon 4 and exons 5 to 7) is connected to the helical domain (encoded by the 3' portion of exon 2, exon 3, and 5' portion of exon 4) by the linker 1 (L1) and linker 2 (L2) peptides. The locations of the P‐loop (P) (red line), three flexible switch regions (S1 to S3) (red line), and the interdomain interface (comprising portions of the α1, αF, α5, β1, P‐loop, and L1 peptide motifs) (blue line) are shown below the *GNA11* exons. The previously reported loss‐of‐function Leu135Gln and Ile200del mutations^11^ are located in the helical and GTPase domains, respectively, whereas the Thr54Met mutation (bold), identified by this study, is located at the interdomain interface. Coding regions are shaded gray and untranslated regions are represented by open boxes.

Familial hypocalciuric hypercalcemia (FHH) is characterized by lifelong elevations of serum calcium concentrations in association with normal or mildly raised serum parathyroid hormone (PTH) concentrations in 80% of patients and low urinary calcium excretion (urinary calcium‐to‐creatinine clearance ratio <0.01) in 80% of patients.^1^, ^2^ FHH may be inherited as an autosomal dominant condition, and it is a genetically heterogeneous disorder with three recognized variants, FHH1‐3. FHH1 (OMIM #145980) is caused by loss‐of‐function mutations of the calcium‐sensing receptor (CaSR), a G‐protein coupled receptor (GPCR)^3^ that initiates activation of the G‐protein subunit αq/11 (Gα_q/11_) family, leading to enhancement of phospholipase C (PLC) activity^4^ and elevation of inositol 1,4,5‐trisphosphate (IP_3_) with rapid increase in intracellular calcium (Ca^2+^_i_) concentrations.^5^, ^6^ These signal transduction events allow the parathyroid CaSR to respond to small fluctuations in the prevailing extracellular calcium concentration ([Ca^2+^]_o_) by inducing alterations in PTH secretion through mechanisms that likely involve effects on PTH mRNA stability^7^ and PTH granule exocytosis from the apical pole of parathyroid cells.^8^ Moreover, the kidney CaSR is considered to influence urinary calcium excretion by modulating expression of claudin proteins that mediate the paracellular reabsorption of calcium in the renal thick ascending limb.^9^, ^10^ FHH2 (OMIM #145981) is the result of loss‐of‐function mutations in the G‐protein subunit‐α11 (Gα_11_), encoded by *GNA11*, and to date only two FHH2‐associated Gα_11_ missense mutations have been reported (Fig. 1).^11^ These two FHH2‐causing Gα_11_ mutations comprise a Leu135Gln missense substitution and an in‐frame isoleucine deletion at codon 200 (Ile200del), which are located in the Gα‐subunit helical and GTPase domains, respectively.^11^ Both of these FHH2‐causing Gα_11_ mutations, which are predicted to disrupt G‐protein activation, have been shown to impair CaSR signal transduction.^11^ FHH3 (OMIM #600740) is caused by loss‐of‐function mutations of the adaptor protein‐2 σ‐subunit (AP2σ), encoded by the *AP2S1* gene. AP2σ has a pivotal role in clathrin‐mediated endocytosis of GPCRs such as the CaSR, and to date more than 50 FHH3 patients with AP2σ mutations, which are all missense mutations involving the Arg15 residue (Arg15Cys, Arg15His and Arg15Leu), have been reported.^12^, ^13^, ^14^, ^15^, ^16^ Approximately 65% of FHH patients will have a CaSR mutation, 5% an AP2σ mutation, <1% a Gα_11_ mutation, and the remaining ∼30% of FHH patients are considered to have involvement of a genetic abnormality that remains to be identified. Here we report the identification of a novel Gα_11_ mutation in a patient with FHH in whom CaSR and AP2σ mutations had been previously excluded.

## Materials and Methods

### Case report

The patient, a 65‐year‐old woman of Indian origin, presented with poor mobility and recurrent falls. She underwent investigation and was found to have hypercalcemia (serum adjusted‐calcium concentration = 2.77 mmol/L, normal range = 2.20–2.60 mmol/L) in association with normal serum concentrations of phosphate (0.85 mmol/L; normal range = 0.70–1.40 mmol/L), creatinine (45 µmol/L; normal range = 40–130 µmol/L), PTH (5.9 pmol/L; normal range = 1.0–7.0 pmol/L), and serum 25‐hydroxyvitamin D (52 nmol/L; normal >50 nmol/L). Urinary calcium‐to‐creatinine clearance ratio was low at 0.01 (normal >0.02). These findings are consistent with a diagnosis of FHH, although there appeared to be an absence of a family history of hypercalcemia, based on the patient not having knowledge of any relatives who suffered from symptomatic hypercalcemia and the relatives not being available for medical assessment. DNA sequence analyses of the *CASR* and *AP2S1* genes had not identified any abnormalities. Informed consent was obtained for the study using protocols approved by the Multi‐Centre Research Ethics Committee (UK) (MREC/02/2/93).

### DNA sequence analysis

DNA sequence analyses of *GNA11* exons 1–7 and their adjacent splice sites (NM_002067) (Fig. 1) was performed using leucocyte DNA and gene‐specific primers (Sigma‐Aldrich, St. Louis, MO, USA), as previously reported.^11^ Publicly accessible databases, including dbSNP (http://www.ncbi.nlm.nih.gov/projects/SNP/), 1000 genomes (http://browser.1000genomes.org), the National Heart, Lung and Blood Institute (NHLBI) Exome Sequencing Project (http://evs.gs.washington.edu/EVS/, EVS data release ESP6500SI) representing the exomes of approximately 6500 individuals, and the Exome Aggregation Consortium (ExAC) (exac.broadinstitute.org) representing exomes of 60,706 unrelated individuals, were examined for the presence of sequence variants, and any potential pathogenic sequence abnormality identified within the patient DNA was confirmed by restriction endonuclease analyses, as described.^12^

### Protein sequence alignment and three‐dimensional modeling of Gα_11_ structure

Protein sequences of Gα_11_ paralogs were aligned using ClustalOmega (http://www.ebi.ac.uk/Tools/msa/clustalo/).^17^ Gα_11_ three‐dimensional modeling was undertaken using the reported three‐dimensional structure of Gα_q_ in complex with the small molecule inhibitor YM‐254890 (Protein Data Bank accession no. 3AH8).^18^ The Gα_q_ protein, which shares 90% identity at the amino acid level with Gα_11_,^11^ was used because crystal structures of Gα_11_ are not available. Molecular modeling was performed using The PyMOL Molecular Graphics System (Version 1.2r3pre, Schrödinger, LL Pymol).^11^

### Cell culture and transfection

Wild‐type and mutant *GNA11* (pBI‐CMV2‐*GNA11*) expression constructs were generated as described,^11^ and transiently transfected into HEK293 cells stably expressing CaSR (HEK293‐CaSR)^12^ using Lipofectamine 2000 (Life Technologies, Carlsbad, CA, USA). The bidirectional pBI‐CMV2 cloning vector was used because it facilitated the co‐expression of Gα_11_ and GFP,^11^, ^12^ and site‐directed mutagenesis was used to generate the mutant *GNA11* construct using the Quikchange Lightning Site‐directed Mutagenesis kit (Agilent Technologies, Santa Clara, CA, USA) and gene‐specific primers (Sigma‐Aldrich), as described.^19^ Cells were maintained in DMEM‐Glutamax media (Thermo‐Fisher, Waltham, MA, USA) with 10% fetal bovine serum (Gibco, Thermo‐Fisher) and 400 μg/mL geneticin (Thermo‐Fisher) at 37°C, 5% CO_2_. Successful transfection was confirmed by visualizing GFP fluorescence using an Eclipse E400 fluorescence microscope with a Y‐FL Epifluorescence attachment and a triband 4,6‐diamidino‐2‐phenylindole‐FITC‐Rhodamine filter, and images captured using a DXM1200C digital camera and NIS Elements software (Nikon, Tokyo, Japan).^11^, ^12^, ^13^ The expression of Gα_11_ and CaSR proteins was also determined by Western blot analyses using anti‐Gα_11_ (Santa Cruz Biotechnology, Dallas, TX, USA), anti‐GFP (Santa Cruz), anti‐calnexin (Millipore, Billerica, MA, USA) or anti‐CaSR (AbCam, Cambridge, UK) antibodies. The Western blots were visualized using an Immuno‐Star Western C kit (Bio‐Rad, Hercules, CA, USA) on a Bio‐Rad Chemidoc XRS+ system.^11^

### Intracellular calcium measurements

The Ca^2+^_i_ responses of HEK293‐CaSR cells expressing wild‐type or mutant Gα_11_ proteins were assessed by a flow cytometry‐based assay, as reported.^11^, ^12^, ^13^ In brief, HEK293‐CaSR cells were cultured in T75 flasks and transiently transfected 24 hours later with 16 μg DNA.^11^ Forty‐eight hours after transfection, the cells were detached, resuspended in calcium (Ca^2+^)‐ and magnesium (Mg^2+^)‐free Hanks' buffered saline solution (HBSS), and loaded with 1 μg/mL Indo‐1‐acetoxymethylester (Indo‐1‐AM) for 1 hour at 37°C. After removal of free dye, cells were resuspended in Ca^2+^‐ and Mg^2+^‐free HBSS and maintained at 37°C. Transfected cells, in suspension, were stimulated by sequentially adding Ca^2+^ to the Ca^2+^‐ and Mg^2+^‐free HBSS to increase the [Ca^2+^]_o_ in a stepwise manner from 0 to 15 mM and then analyzed on a MoFlo modular flow cytometer (Beckman Coulter, Indianapolis, IN, USA) by simultaneous measurements of GFP expression (at 525 nm), Ca^2+^_i_‐bound Indo‐1AM (at 410 nm), and free Indo‐1AM (ie, not bound to Ca^2+^_i_) (at 485 nm), using a JDSU Xcyte UV laser (Coherent Radiation, Santa Clara, CA, USA), on each cell at each [Ca^2+^]_o_, as described^11^, ^12^. The peak mean fluorescence ratio of the Ca^2+^_i_ transient response after each individual stimulus was measured using Cytomation Summit software (Beckman Coulter) and expressed as a normalized response, as described.^11^, ^12^ Nonlinear regression of concentration‐response curves was performed with GraphPad Prism (GraphPad, La Jolla, CA, USA) using the normalized response at each [Ca^2+^]_o_ for each separate experiment for the determination of EC_50_ (ie, [Ca^2+^]_o_ required for 50% of the maximal response) and Hill coefficient values. The maximal signaling response was measured as a fold‐change of the peak transient Ca^2+^_i_ response to the basal Ca^2+^_i_ response measured at 0 mM [Ca^2+^]_o_. The maximal signaling responses for mutant Gα_11_ proteins were expressed as a percentage of the wild‐type Gα_11_ protein maximal signaling response. The mean EC_50_ and Hill coefficients obtained from four separate transfection experiments were used for statistical comparison by using the *F*‐test, and alterations in maximal signaling responses assessed using the Mann‐Whitney *U* test.

## Results

### Identification of a novel Thr54Met Gα_11_ mutation in an FHH proband

**Figure 2:**
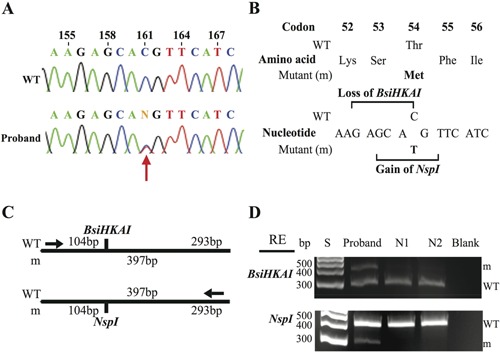
Identification of a Thr54Met *GNA11* mutation in a FHH proband. (*A*) DNA sequence analyses revealed a heterozygous C‐to‐T transition at nucleotide c.161 (red arrow) within exon 2 of *GNA11*. (*B*) This sequence abnormality was predicted to lead to a missense amino acid substitution of Thr to Met at codon 54, resulting in the loss of a *BsiHKAI* restriction endonuclease site (GAGCA/C) and gain of an *NspI* (GCATG/T) restriction endonuclease site. (*C*) Restriction maps showing that *BsiHKAI* digestion would result in two products of 104 bp and 293 bp from the wild‐type (WT) sequence but not the mutant (m) sequence. In contrast, *NspI* digestion results in two products of 104 bp and 293 bp from the m sequence but not the WT sequence. (*D*) Restriction endonuclease (RE) digest of PCR products of exon 2 of *GNA11* demonstrating the heterozygous C‐to‐T transition and confirming its absence in two unrelated unaffected individuals (N1 and N2). S = size marker. For *BsiHKAI* RE digest the mutant product (397 bp) and WT product (293 bp) are shown; the WT product at 104 bp is not shown. For *NspI* RE digest the WT product (397 bp) and mutant product (293 bp) are shown; the mutant product at 104 bp is not shown.

**Figure 3:**
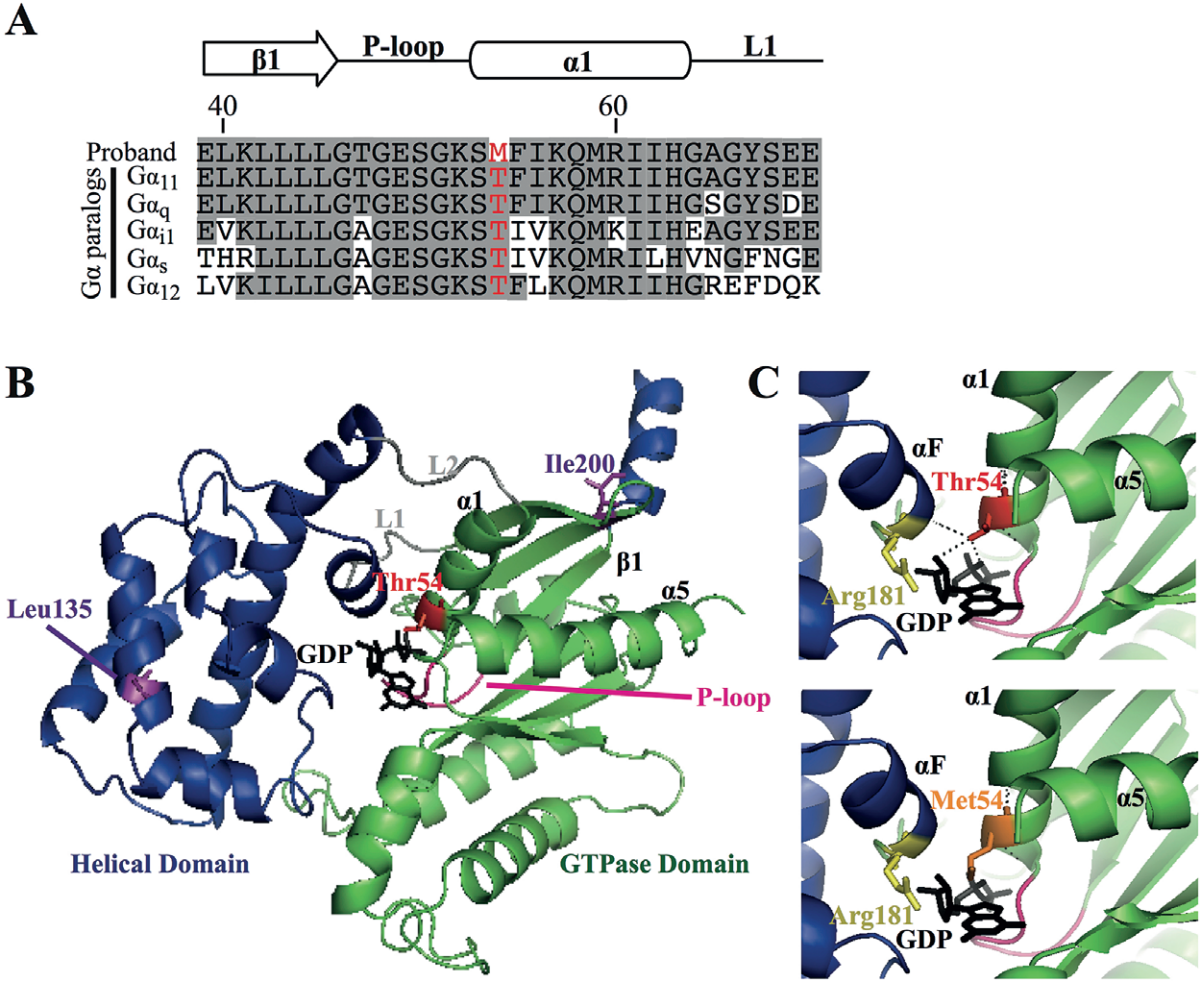
Predicted effects of the Thr54Met mutation on the Gα_11_ protein. (*A*) Multiple protein sequence alignment of residues contributing to the interdomain interface of Gα‐subunit paralogs. The wild‐type Thr54 (T) and mutant Met54 (M) residues are shown in red. Conserved residues are shaded gray. (*B*) Overall three‐dimensional structure of the Gα_11_ protein. The helical (blue) and GTPase (green) domains are connected by the L1 and L2 peptides (gray). The GTPase domain contains the P‐loop (pink), which binds GDP (black) at the interdomain interface (comprising parts of α1, α5, αF, P‐loop, L1, and β1 peptide motifs). The reported Leu135Gln and Ile200del mutations^11^ are shown in purple, and the Thr54Met mutation is shown in red. (*C*) Close‐up views of the Gα_11_ interdomain interface, which show the wild‐type Thr54 residue, located in the GTPase domain, form polar contacts (hatched black lines) with GDP and the Arg181 residue (yellow) of the helical domain αF helix. Substitution of the wild‐type Thr54 residue with the mutant Met54 residue (orange) is predicted to disrupt these polar contacts, thereby impairing interdomain interactions and the binding of GDP.

DNA sequence analyses of the *GNA11* coding regions and adjacent splice sites (Fig. 1) identified a heterozygous C‐to‐T transition at nucleotide c.161, in the FHH patient (Figure 2A). This C‐to‐T transition (ACG to ATG) resulted in a missense substitution, Thr54Met, of the Gα_11_ protein (Fig. 2 *B*). The sequence alteration also led to the gain of an *Nsp*I and loss of a *BsiHKAI* restriction endonuclease site (Fig. 2 *B*), which were used to confirm the presence of the mutation in the patient (Fig. 2 *C, D*). Bioinformatic analyses using SIFT and MutationTasting software^20^, ^21^ predicted the variant to be damaging and likely disease‐causing (SIFT score 0, MutationTasting score 0.99). In addition, the absence of this DNA sequence abnormality in >6500 exomes from the NHLBI‐ESP cohort and >60,700 exomes from the ExAC cohort, together with evolutionary conservation of the Thr54 residue in vertebrate Gα‐subunit paralogs (Fig. 3 *A*), indicated that the Thr54Met abnormality likely represented a pathogenic *GNA11* mutation rather than a benign polymorphic variant.

### Structural characterization of the Thr54Met Gα_11_ mutant protein

The Thr54Met mutation is located within the Gα_11_ α‐1 helix (Fig. 1 and Fig. 3 *A, B*), which comprises part of the interface at which the GTPase and helical domains interact to bind GDP and GTP.^22^ In contrast, the previously reported FHH2‐causing Iledel200 and Leu135Gln mutations,^11^ which affect the GTPase and helical domains of Gα_11_, respectively, are situated away from the guanine‐nucleotide binding site (Fig. 1 and Fig. 3 *B*). The Thr54 Gα_11_ residue is located next to the phosphate‐binding loop (P‐loop) (Fig. 3 *A*), which is a highly conserved nucleotide‐binding peptide motif that plays a critical role in binding GDP.^23^, ^24^, ^25^ Three‐dimensional homology modeling of the Gα_11_ protein revealed the wild‐type Thr54 residue to form polar contacts with the ribose and β‐phosphate moieties of GDP within the interdomain interface (Fig. 3 *B, C*) and to interact with the α‐F helix of the helical domain, which also mediates GDP binding (Fig. 3 *C*).^22^ These findings are consistent with the reported role of the α‐1 helix as a structural hub that mediates interactions between the GTPase and helical domains to ensure GDP binding, thereby maintaining the Gα‐subunit in an inactive conformation.^26^ The Gα_11_ Met54 mutant is predicted to disrupt these interdomain contacts and alter GDP binding (Fig. 3 *C*).

### Functional characterization of the Thr54Met Gα_11_ mutant protein

**Figure 4:**
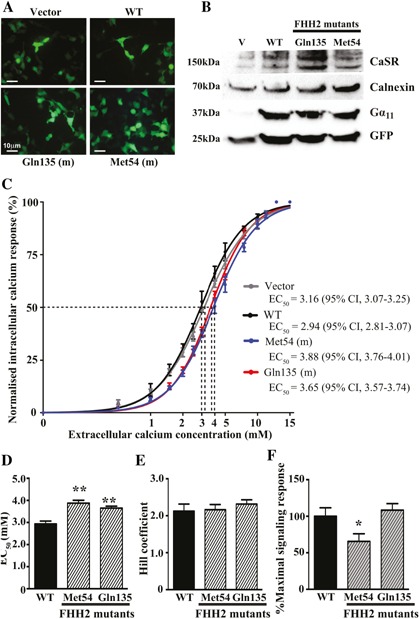
Functional characterization of wild‐type and FHH2‐associated mutant Gα_11_ proteins. (*A*) Fluorescence microscopy of HEK293 cells stably expressing CaSR (HEK293‐CaSR) and transiently transfected with wild‐type (WT) or FHH2‐associated mutant (Met54 and Gln135) pBI‐CMV2‐*GNA11*‐GFP constructs, or with vector only. GFP expression in these cells indicates successful transfection and expression by these constructs. Scale bar = 10 µm. (*B*) Western blot analyses of whole‐cell lysates using antibodies to CaSR, calnexin, Gα_11_, and GFP. Transient transfection of WT or FHH2‐associated mutant constructs resulted in overexpression of Gα_11_ when normalized to calnexin expression. (*C*) Concentration‐response curves showing normalized Ca^2+^_i_ response to changes in [Ca^2+^]_o_ of HEK293‐CaSR cells transfected with WT or FHH2‐associated Gα_11_ mutants. The Ca^2+^_i_ responses are shown as the mean ± SEM of 4 independent transfections. The FHH2‐associated Gα_11_ mutants (Met54 and Gln135) led to a rightward shift of the concentration‐response curves (blue and red, respectively) when compared with WT Gα_11_ (black), which harbors Thr and Leu residues at codons 54 and 135, respectively. (*D*) The Met54 and Gln135 mutants (shaded bars) were associated with significantly increased EC_50_ values compared with cells expressing WT Gα_11_ (black bar). (*E*) The Hill coefficients of the wild‐type and mutant Gα_11_ proteins were similar (ie, not significantly different). (*F*) The Met54 mutant was associated with a significantly reduced maximal signaling response compared with WT and mutant Gln135 Gα_11_ proteins. *p < 0.05, **p < 0.01.

To determine the effects of the predicted changes in Gα_11_ structure (Fig. 3 *B, C*) on CaSR‐mediated signaling, Ca^2+^_i_ responses to alterations in [Ca^2+^]_o_ were assessed in HEK293‐CaSR cells that were transiently transfected with either the pBI‐CMV2 empty vector or pBI‐CMV2 expressing the wild‐type (Thr54) or mutant (Met54) Gα_11_ proteins. The Ca^2+^_i_ responses of cells expressing the Met54 Gα_11_ mutant were also compared with cells transiently transfected with the reported FHH2‐associated Gln135 Gα_11_ mutant protein.^11^ Expression of CaSR, Gα_11_ and GFP was confirmed by fluorescence microscopy and/or Western blot analyses (Fig. 4 *A, B*). Calnexin was used as a loading control in Western blot analyses, and Gα_11_ expression was demonstrated to be similar in cells transiently transfected with wild‐type or mutant Gα_11_ proteins and greater than that of cells transfected with the empty pBI‐CMV2 vector (Fig. 4 *B*). The Ca^2+^_i_ responses in wild‐type and mutant Gα_11_‐expressing cells were shown to increase in a dose‐dependent manner after stimulation with increasing concentrations of Ca^2+^_o_ between 0–15 mM. However, exposure to a significantly greater [Ca^2+^]_o_ was required to achieve half‐maximal (EC_50_) Ca^2+^_i_ responses for cells expressing either the Met54 or Gln135 mutant Gα_11_ proteins compared with wild‐type‐expressing cells. (Fig. 4 *C, D*). Thus, the Met54 or Gln135 mutant‐expressing cells showed rightward shifts in the concentration‐response curves, with significantly elevated mean EC_50_ values (*p* < 0.01) of 3.88 mM (95% confidence interval [CI] 3.76–4.01 mM) and 3.65 mM (95% CI 3.57–3.74 mM), respectively, compared with 2.94 mM (95% CI 2.81–3.07 mM) for wild‐type expressing cells and consistent with the Gα_11_ mutants leading to an impairment of CaSR signal transduction (Fig. 4 *C, D*). The Hill coefficients did not significantly differ between wild‐type and mutant Gα_11_‐expressing cells (Fig. 4 *E*). However, cells expressing the Met54 mutant had significantly reduced maximal signaling responses compared with cells expressing either wild‐type or Gln135 mutant Gα_11_ proteins (*p* < 0.05) (Fig. 4 *F*).

## Discussion

Our studies have identified a novel heterozygous germline *GNA11* mutation in a patient with FHH, which resulted in an impairment of Ca^2+^_i_ signaling similar to the loss‐of‐function previously reported for the FHH2‐associated Leu135Gln and Ile200del *GNA11* mutations.^11^ The Thr54Met mutation represents only the third loss‐of‐function *GNA11* mutation to be reported, and thus these findings provide further support for a critical role of the Gα_11_ protein in parathyroid gland function and Ca^2+^_o_ homeostasis, and highlight the importance of *GNA11* gene analyses in FHH patients that do not harbor *CASR* or *AP2S1* mutations. The Thr54Met, Leu135Gln, and Ile200del loss‐of‐function *GNA11* mutations are all associated with a mild FHH phenotype characterized by serum adjusted‐calcium concentrations <2.80 mmol/L, and these clinical findings are in keeping with our in vitro studies that have shown FHH2‐associated mutations to induce only minor disturbances of CaSR signal transduction.^11^ Indeed, the FHH2 mutants were associated with around a 30% increase in the EC_50_ values of HEK293‐CaSR cells used in this study, whereas CaSR mutations leading to FHH1 generally cause a >50% increase in the EC_50_ value.^3^, ^27^ The milder shift in the Ca^2+^_o_ set point of cells expressing FHH2‐associated Gα_11_ mutants indicates that the CaSR may promote Ca^2+^_i_ signaling by Gα_11_‐independent mechanisms, such as via the related Gα_q_ protein. Indeed, reported studies that selectively ablated Gα_q_ and/or Gα_11_ in the parathyroid glands of mice have highlighted the importance of both of these G‐proteins for CaSR function,^28^, ^29^ and in the setting of FHH1, CaSR mutations likely impair Ca^2+^_i_ responses via both Gα_11_ and Gα_q_, thus leading to a greater loss‐of‐function than Gα_11_ mutations that cause FHH2.

The Gα_11_‐subunit consists of a Ras‐like GTPase domain that binds GDP and GTP, and a smaller helical domain that acts as a clasp to secure these bound guanine‐nucleotides.^30^ Three‐dimensional modeling indicated the Thr54Met mutation to be located at the interdomain interface, which represents a highly conserved and critical region containing the P‐loop motif that binds GDP^23^, ^24^ and also facilitates interactions between the helical and GTPase domains that maintain Gα‐subunits in an inactive GDP‐bound conformation.^22^ The Thr54Met mutation likely alters GDP binding, but in contrast to the other reported Gα_11_ mutations (Arg60Cys, Arg60Leu, and Arg181Gln),^11^, ^31^, ^32^ which are also located at the interdomain interface (Fig. 3 *B, C*) and cause Gα_11_ gain‐of‐function that is associated with the clinical disorder of autosomal dominant hypocalcemia type‐2 (ADH2), the Thr54Met Gα_11_ mutation causes loss‐of‐function and FHH2. Thus, it seems that mutations involving the Gα_11_ interdomain interface may result in Gα_11_ loss‐of‐function or gain‐of‐function. Crystal structures of Gα_11_ proteins are not available to evaluate the structure‐function effects of the Thr54Met mutation at the interdomain interface; however, the introduction of the mutant Met54 residue may sterically impair G‐protein function, as highlighted by a previous crystallography study of a loss‐of‐function G‐protein alpha‐i (Gα_i_) P‐loop mutation, which revealed the mutant Gα_i_ residue to sterically hinder conformational changes of the flexible “switch” regions during Gα‐subunit activation.^33^ Moreover, interdomain interface mutations that disrupt guanine‐nucleotide binding may also result in an “empty‐pocket” mutant Gα‐subunit that exerts dominant‐negative effects by binding and sequestering partner GPCRs.^34^ These findings may also help to provide an explanation for the observed differences in the maximal signaling responses of the FHH2‐causing Met54 and Gln135 Gα_11_ mutants (Fig. 4 *F*). Thus, the Met54 Gα_11_ mutant, but not the Gln135 Gα_11_ mutant, led to a significant reduction in the maximal signaling response of CaSR‐expressing cells (Fig. 4 *F*), even though both Gα_11_ mutants increase the Ca^2+^_o_ set point of CaSR‐expressing cells to a similar degree, as illustrated by their EC_50_ values (Fig. 4 *D*). The maximal signaling response of a GPCR is influenced by the ability of the receptor to couple with its cognate G‐protein,^35^ and thus it is possible that the Met54 Gα_11_ mutant impairs coupling and/or dissociation of Gα_11_ from the CaSR by influencing guanine‐nucleotide binding at the interdomain interface,^34^ whereas the Gln135 Gα_11_ mutant, which is located in the Gα_11_ helical domain and not predicted to influence CaSR‐Gα_11_ coupling, may potentially diminish CaSR signal transduction by influencing the interaction of Gα_11_ with downstream effectors.^36^

In conclusion, we have identified a novel Gα_11_ loss‐of‐function mutation, Thr54Met, that causes FHH2 and which provides new insights into the critical role of the Gα_11_ interdomain interface in CaSR signaling.

## Disclosures

All authors state that they have no conflicts of interest.
